# Epidemiological Profile of Urinary and Intestinal Schistosomiasis in the Kingdom of Saudi Arabia: A Seven-Year Retrospective Study

**DOI:** 10.3390/tropicalmed9010011

**Published:** 2023-12-29

**Authors:** Rafat Zrieq, Mohamed Ali Alzain, Reem M. Ali, Awfa Y. Alazzeh, Anas O. Tirawi, Rozan Attili, Tolgahan Acar, Najoua Haouas

**Affiliations:** 1 Department of Public Health, College of Public Health and Health Informatics, University of Ha’il, Ha’il 2440, Saudi Arabia; m.alzain@uoh.edu.sa; 2 Applied Science, Research Center, Applied Science Private University, Amman 11931, Jordan; 3 Department of Clinical Laboratory Sciences, Faculty of Applied Medical Sciences, University of Ha’il, Ha’il 2440, Saudi Arabia; re.ali@uoh.edu.sa; 4 Department of Clinical Nutrition, College of Applied Medical Sciences, University of Ha’il, Ha’il 2440, Saudi Arabia; a.alazzeh@uoh.edu.sa; 5 Faculty of Medicine, Yarmouk University, Irbid 21163, Jordan; 2018899055@ses.yu.edu.jo; 6 Department of Medical Laboratory Science, Faculty of Pharmacy and Medical Sciences, Hebron University, Hebron P.O. Box 40, Palestine; rozana@hebron.edu; 7 Department of Physical Therapy, College of Applied Medical Sciences, University of Ha’il, Ha’il 2440, Saudi Arabia; t.acar@uoh.edu.sa; 8 Laboratory of Medical and Molecular Parasitology-Mycology LP3M (Code LR12ES08), Department of Clinical Biology B, Faculty of Pharmacy, University of Monastir, Street 1, Avicenne, Monastir 5000, Tunisia

**Keywords:** schistosomiasis, *S. haematobium*, *S. mansoni*, health facility report, Saudi Arabia

## Abstract

Background: Despite the marked decline of schistosomiasis in Saudi Arabia in recent years, it is still reported in several regions. This study investigates the epidemiology of schistosomiasis in Saudi Arabia over seven years (2014–2020). Methodology: A retrospective study was retrieved from the annual reports of the Ministry of Health. A Geographic Information System GIS, Chi-square, and logistic regression were used to analyze the data. Results: Out of the 4,371,481 tested, 680 cases were positive for schistosomiasis, with a cumulative incidence rate of 2.155/100,000 population. This number showed significant variation over the study period (*p* value < 0.001). The highest number of cases detected in 2015 was almost 2-fold (OR = 1.93; 95%CI: 1.36–2.74) higher than in 2020. Both clinical forms (urinary and intestinal schistosomiasis) exist in Saudi Arabia (79.6% and 20.4% of all schistosomiasis cases, respectively). Schistosomiasis was reported in seven out of thirteen regions. Among them, Mecca has a relatively high number of cases (OR = 5.57; 95%CI: 2.49–12.47). Conversely, the Eastern Province has a low number of cases (OR = 0.09; 95%CI: 0.02–0.39) when compared to the Najran region (*p* value > 0.001). Regarding the distribution of schistosomiasis cases by gender and nationality, we noticed that most of the positive cases were found among males (70.6%) and expatriates (83.6%). Conclusions: The persistence of schistosomiasis and the disparity in the demographic factors underscores the imperative for intensified and integrative One Health interventions to combat this disease in Saudi Arabia.

## 1. Introduction 

Schistosomiasis is a parasitic disease caused by flukes (trematodes) of the genus *Schistosoma* [1]. *Schistosoma* has a complex indirect life cycle. Once the adult worms mate and enter a host’s bloodstream, the females produce eggs that are excreted through human stool or urine. Afterwards, the eggs hatch into miracidia within freshwater habitats and rapidly infiltrate intermediate host snails, where they have the potential to mature into sporocysts and eventually evolve into cercariae through the process of asexual reproduction. Direct contact with cercaria released from infected snails results in the transmission of the infection to humans [2]. Despite Schistosomiasis being one of the oldest parasitic diseases affecting humans, it continues to pose a significant threat to public health in approximately 77 developing countries in the tropical and subtropical regions [3,4]. Although Schistosomiasis is one of the most prevalent human parasitic infections, it is still one of the most neglected tropical diseases (NTDs) [5,6]. Approximately 700 million individuals globally are considered susceptible to infection, with more than 240 million confirmed cases [7,8]. The infection is predominantly concentrated in sub-Saharan Africa, where it accounts for more than 90% of cases and leads to an annual death toll of nearly 300,000 [3,9]. 

Several diagnostic techniques can be applied to the diagnosis of *Schistosoma* infections [10,11]. The primary diagnostic tests for *Schistosoma* eggs are microscopy analyses of feces or urine. The use of monoclonal antibody lateral flow enzyme [12] and enzyme-linked immunosorbent assay (ELISA) has improved the detection of *Schistosoma*, but these assays are still less appropriate in regions where Schistosomiasis is not as common [13].

Various approaches have been used to control *Schistosoma*, such as the first mass drug distribution for adults and children [14]. Additionally, a nationwide initiative was put into place that combined chemotherapy and/or snail control [14].

Although strides have been made in mitigating the disease and decreasing morbidity and mortality rates, schistosomiasis persists in expanding its geographical reach. Introducing of the disease into new areas is primarily attributable to environmental shifts arising from the development of water resources, as well as population growth and migration [15]. The Kingdom of Saudi Arabia (KSA) is one of the largest Arab countries, occupying approximately 80% of the Arabian Peninsula. It is bordered by historical schistosomiasis hotspots, including Yemen, Egypt, and Iraq, all of which have a well-documented history of human schistosomiasis [16,17,18]. Schistosomiasis was first documented in Saudi Arabia in 1887 through Muslim pilgrims returning from Mecca but is believed to have been endemic since the 10th century [19]. Following its initial detection, many *S. haematobium* and *S. mansoni* cases were documented throughout the country [20], and it was suspected that the disease was endemic across the whole region except the Eastern Province, Qassim, and Empty Quarter Provinces [21]. The first comprehensive analysis of the disease and snail distribution was carried out in 1967, with an estimated countrywide prevalence of 17%. However, subsequent surveys suggest that the initial estimates may have been inflated considering the relative absence of disease in urban centers and the Eastern region, in addition to the focal nature of the disease in rural areas [17,20]. Concerning the intermediate host snails for *Schistosoma*, two genera were described in Saudi Arabia: *Bulinus* and *Biomphalaria*. In 1976, Arfaa described the snail *Biomphalaria arabica* as the intermediate host for *S. mansoni* in Saudi Arabia [17]. Over a decade later, three snail’s species, *Bulinus wright*, *Bulinus beccarii* and *Bulinus truncates*, were incriminated as intermediate hosts for *S. haematobium* [22]. In 2008, Bin Dajem assessed the trematode infection of freshwater snails collected in 15 localities of Aseer region, South Saudi Arabia; however, no infection was detected [23].

*Schistosoma mansoni* infections were primarily documented in the highlands of western regions, along with specific areas in the Central and Northern Regions. *Schistosoma haematobium* infections were mainly reported in Tihama Assir and the lowland coastal plain in the southern regions [24]. Although cases of human infection with *Schistosoma japonicum* were detected among some expatriate residents, the parasite failed to complete its life cycle in the country [25].

Several surveys have been carried out in different regions across KSA including Abha district [26], Riyadh [27], AL-Khobar [28], Mecca [29] and Al-Baha [30]. Nevertheless, no study has been conducted to assess the schistosomiasis status throughout the kingdom. Consequently, there has been no published data regarding the epidemiological characteristics of schistosomiasis across all provinces in Saudi Arabia. Hence, our study aims to investigate the epidemiology features of schistosomiasis in the kingdom of Saudi Arabia and to assess its temporal evolution during a period of 7 years (2014–2020) at the national level.

## 2. Materials and Methods

### 2.1. Study Area

Saudi Arabia is located in Western Asia, with a land area of approximately 2,150,000 km^2^, making it the fifth-largest geographical state in Asia [31]. It shares borders with Jordan and Iraq in the north, Kuwait in the northeast, Qatar, Bahrain, and the United Arab Emirates in the east, Oman in the southeast, and Yemen in the south [31]. Saudi Arabia has a total population of 28.7 million, of which 20 million are Saudi nationals and approximately 8 million are expatriates [25,32].

Saudi Arabia’s geography is dominated by the Arabian Desert, associated with semi-desert, shrubland, and several mountain ranges and highlands [31]. There are a few lakes in the country but no permanent rivers. The main topographical feature is the central plateau that rises abruptly from the Red Sea and gradually descends into the Nejd [31]. 

In terms of climate, Saudi Arabia is generally characterized by a desert climate, except for the south-western province of Asir. Daytime temperatures are very high, with a sharp temperature drop at night. Average summer temperatures are approximately 45 °C but can be as high as 54 °C. In the winter, the temperature rarely drops below 0 °C. In the spring and autumn, the weather is temperate; averaging approximately 29 °C. Annual rainfall is extremely low [27,33].

### 2.2. Study Design and Period

A retrospective case series study was conducted to investigate data on human schistosomiasis in KSA; data were collected over seven years (2014–2020). 

### 2.3. Data Collection

Data on schistosomiasis-diagnosed cases (age, gender, nationality, and type of schistosomiasis) were collected from the Ministry of Health reports (MOHSA, statistical books, 2014–2020). The reports were obtained from the official website of the General Authority for Statistics (GASTAT), which contains official statistical data collected from various health facilities. The sources of these reports are from the schistosomiasis surveillance program in KSA. Schistosomiasis is considered one of the infectious diseases that must be reported within a week to the Ministry of Health through surveillance programs. Once a suspected case arises in a healthcare facility, a microscopic stool and/or urine examination is taken for laboratory diagnosis. A confirmed case is defined as a positive microscopy examination. 

### 2.4. Data Analysis

The data were extracted from the General Authority for Statistics (GASTAT) on an Excel spreadsheet. Subsequently, the data were processed using Statistical Package for Social Sciences (SPSS) version 28. A Geographic Information System (GIS) was utilized to map the geographical distribution of schistosomiasis during the study period. We weighed cases by the frequency of examined cases and then by the number of positive cases for statistical analysis. The Chi-square test was employed to visualize the distribution of cases across different years (2014–2020), age groups, affected regions, gender, and types of schistosomiasis. Furthermore, logistic regression was used to predict positive cases across different years and regions, with the year 2020 and Najran serving as references. A two-tailed *p*-value < 0.05 was considered statistically significant.

## 3. Results

### 3.1. Incidences and Distribution of Schistosoma Cases in KSA 2014–2020

Over the seven-year period (2014–2020), a total of 4,371,481 individuals sought schistosomiasis diagnosis at various Saudi healthcare facilities. This group included suspicious schistosomiasis cases and asymptomatic expatriates who were diagnosed with schistosomiasis in the context of routine and systematic medical check-up to ensure that they were infection-free. Among them, 680 were diagnosed with either intestinal or urinary schistosomiasis, corresponding to an overall of 0.0155% of the diagnosed cases during the study period (Table 1). Chi-square test analysis indicated a significant fluctuation in the distribution of *Schistosoma* infections throughout this seven-year investigation. The temporal analysis demonstrated significant variations in the distribution of schistosomiasis across the study period, as confirmed by the chi-square test. Interestingly, by using the year 2020 as a reference in binary regression, we noticed that the number of positive schistosomiasis cases was approximately twice as high as in 2020 (OR = 1.93; 95%CI: 1.36–2.74). In a close matter, for the period 2016–2018, the likelihood of positive cases was more than 1.5-fold higher compared to 2020 (OR = 1.56; 95%CI: 1.08–2.23), (OR = 1.65; 95%CI: 1.14–2.38), and (OR = 1.55; 95%CI: 1.07–2.25), respectively (Table 1). 

**Figure 1:**
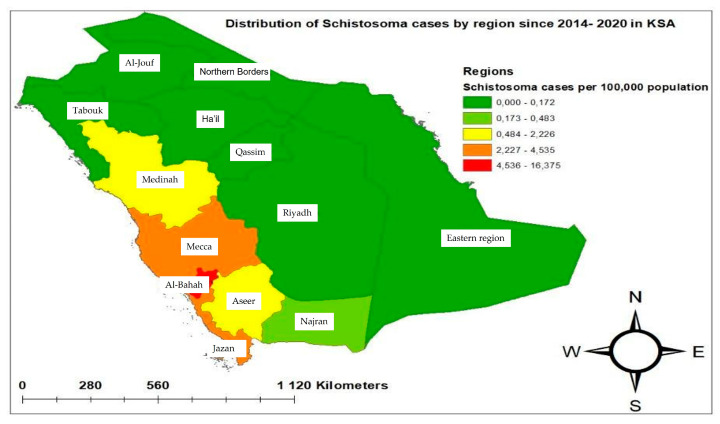
Geographical distribution of schistosomiasis in KSA by regions from 2014 to 2020.

The spatial analysis of the distribution of schistosomiasis cases across KSA regions was investigated using GIS. Strikingly, most of the schistosomiasis cases clustered in the western and south-western regions along the Red Sea coast. Notably, schistosomiasis was clearly absent in central and northern regions such as Riyadh, Hail, Tabouk, and Al-Jouf, as shown in Figure 1.

The overall cumulative incidence over the study period was 2.56/per 100,000 population. Indeed, among the 13 investigated regions, Mecca has a significantly higher number of schistosomiasis cases, being more than 5 fold (OR = 5.57; 95%CI: 2.49–12.47) as likely to have cases compared to the Najran region (Table 2 and Figure 1). Following closely were the Al-Bahah and Aseer regions which had more than 3 fold the likelihood of cases compared to the Najran region, (OR = 2.49; 95%CI: 1.09–5.69) and (OR = 2.29; 95%CI: 0.98–5.23), respectively. In contrast, the Eastern region was less likely to have schistosomiasis infection cases (OR = 0.09; 95%CI: 0.02–0.39) than the Najran region. 

### 3.2. Clinical Types of Schistosomiasis and Their Distribution among KSA Regions (2014–2020)

Both types of schistosomiasis (intestinal and urinary) exist in Saudi Arabia (Table 3). Intestinal schistosomiasis was the most significant common clinical form, accounting for 79.6% of cases (*p* value < 0.001). Most of the positive recorded cases in Mecca, Al-Bahah, and Medinah were intestinal schistosomiasis. In contrast, most positive cases in Jazan, Aseer, Najran, and the Eastern regions were urinary schistosomiasis (*p* value < 0.001).

### 3.3. Gender Disparities in Schistosomiasis Distribution across Regions in KSA (2014–2020)

Having analyzed the distribution of positive schistosomiasis cases according to gender, we noticed that both genders were affected by schistosomiasis in Saudi Arabia (Table 4). Remarkably, the most significant common cases were males (n = 584, 85.9%), (*p* value < 0.001). Further, schistosomiasis cases were dominant for male in all regions. Interestingly, all cases in Najran were males.

### 3.4. Nationality-Based Distribution of Schistosomiasis across Regions in KSA (2014–2020)

Among the 680 reported schistosomiasis cases, 200 positive cases (29.4%) were Saudi versus 480 positive cases (70.6%) were expatriates (Table 5), reflecting a divergence in the prevalence patterns among these groups. The Chi-square test revealed that the percentage of the expatriates’ positive cases was significantly higher than the Saudis, especially in Mecca, Medinah, Aseer, Najeran, and Jazan regions (*p* value < 0.001). In contrast, the percentage of Saudi positive cases in Al-Bahah and the Eastern region were significantly higher than in expatriates (*p* value < 0.001). 

### 3.5. Age-Based Distribution of Schistosomiasis across Regions in KSA (2014–2020)

The chi-square test revealed a statistically significant difference in the distribution of cases among different age groups in affected regions (Table 6). More than three-quarters (75.5%) of the cases were in the 15–39 year age group 15–39 yars, followed by 15.4% of the cases in the 5–14 year age group (*p* value < 0.001). Less positive cases were reported in the older people (>40 years), while only one case in the <5 year age group was reported in the Aseer region. Hint, schistosomiasis was dominant within the 15–39 year age group in all regions of KSA. 

## 4. Discussion

Despite the marked decline of schistosomiasis in KSA, our study revealed that schistosomiasis still persists. During the study period (2014–2020), more than 4 million individuals from all over the Kingdom were examined, out of which 680 positive cases with a cumulative incidence rate of 2.56/100,000 population were reported. Temporal analysis revealed that the number of schistosomiasis cases significantly decreased through the period 2014–2020 (*p* value < 0.001), suggesting positive progress in controlling schistosomiasis infection in KSA. Remarkably, the incidence of schistosomiasis through 2014–2020 is lower than those previously reported in KSA, from 1.7% in 2002 to 0.7% in 2003 and to 0.6% in 2004 [4]. In general, the marked decline of schistosomiasis in KSA was most likely the result of the adoption of an effective program for the elimination of schistosomiasis later in the past century [4,34].The schistosomiasis elimination program has continued successfully in recent years, with a decrease in the infection rate of more than 75% since 2015. The schistosomiasis control program included early case detection and widespread chemotherapy for high-risk groups, especially those younger than 40 years (>90% of the cases), improved sanitation, health education, snail control and environmental management. 

Despite these efforts to control and prevent this disease, challenges persist due to many factors, such as water resource development, agricultural practices and the large influx of foreign workers from endemic countries, e.g., Sudan, Yemen, Egypt and India. According to the WHO 2012, KSA was classified as a “country requiring updating for planning and implementation purposes”. Thus, ongoing surveillance and scientific research investigations are crucial to continuously follow-up on the prevalence of schistosomiasis, to identify vulnerable groups and to gain insights into the distribution and risk factors of the disease. These data are crucial for developing evidence-based interventions, implementing preventive measures and optimizing resource allocation for effective and sustainable control interventions in the country. By aligning with the WHO’s road map for neglected tropical diseases (NTDs) 2021–2030, Saudi health authorities and policy makers must continue consolidating efforts, increasing coordination and scaling-up innovative approaches to address the challenges of schistosomiasis. It is important to highlight that the objectives of the National 2030 Saudi Arabia Vision are closely aligned with the WHO’s new NTDs road map. Indeed, the 2030 Saudi Arabia Vision focuses on the transformation of the healthcare sector by prioritizing innovation, financial sustainability and disease prevention. 

Moreover, considering the long lifespan of the parasite, a part of the reported schistosomiasis cases in KSA in this study could most likely be as post-transmission infections due to imported infection by immigrant movements from endemic countries [35,36]. Indeed, it was demonstrated that untreated individuals can harbor live schistosomiasis for 3 to 4 years for *S. haematobium* and for 5.7 to 23 years for *S. mansoni* [37,38]. This expectation is consistent with our results as 70.6% of the positive cases were from expatriates. Indeed, approximately 36% of the Saudi population are expatriates and the kingdom receives more than 8 million pilgrims per year [34]. The majority of the expatriates came from endemic countries, e.g., Sudan, Yemen, Egypt and India. In this context, it has been estimated that ten million *Schistosoma*-infected individuals in the Middle East and North Africa (MENA) countries are clustered in Egypt and Yemen [7] which strongly support our expectation. 

Spatial analysis revealed that schistosomiasis exists in seven out of the thirteen regions of KSA. Schistosomiasis is reported in the western and the south-western regions of the country including Mecca, Al-Bahah, and Jazan as well as, albeit to a lesser extent, the Eastern region, Medinah and Najran. Remarkably, schistosomiasis was absent in the central and northern regions. Our results are in good alignment with the historical data. In 2005, Saudi regions were divided into three distinct endemic categories. The first category included four schistosomiasis-free locations that are Riyadh, Hail, Tabuk, and Al-Jouf. The second category included low endemicity areas, encompassing Madinah, Najran, Makkah, Jeddah, and Al-Taif, whereas the third category, including Jizan, Baha, Aseer, and Bisha, was categorized as high endemicity areas. Collectively, this suggests that Mecca had less response to the initiated national program for the elimination of the disease in 2005 than other regions. Moreover, Jazan and Al-Baha are still among the high endemicity areas [21]. It is important to highlight that Mecca is the main destination for pilgrims, while some visit Medina. The absence of studies on the different *Schistosoma* life cycle components and their contribution to maintaining of parasite transmission in Saudi Arabia prevent us from concluding the origin of the reported schistosomiasis cases. More investigations on the ecological niches, environmental, and climatic features affecting snails’ populations are needed in the Saudi *Schistosoma* foci better to understand the real situation of schistosomiasis in the country. Studies assessing the spatio-temporal evolution of schistosomiasis in the reported Saudi areas are primordial to draw the parasite circulation profile and thereby to implement an evidence-based control strategy. 

Moreover, our data revealed that both clinical forms of schistosomiasis (intestinal and urinary) are found in KSA. Most positive cases in Mecca, Al-Bahah, and Medinah were intestinal schistosomiasis. In contrast, urinary schistosomiasis is more prevalent in Jazan, Aseer, Najran, and Eastern regions. These distinct profiles reflect the complex ecology of *schistosoma* species depending on specific snail species, climatic conditions and human behavior. Unfortunately, malacological studies related to *Schistosoma* are scarce in Saudi Arabia. Except the study of Bin Dajem, 2009 which assessed trematode infection of collected freshwater snails in Asser region, no studies were carried out to assess the distribution, the ecology and the population dynamics of snails. Data are also lacking concerning snail behavior, habitat preferences and the impact of environmental changes on snail habitats. This knowledge gap prevents implementing integrated control strategies focusing on intermediate host snail population.

This study’s finding of a higher incidence of schistosomiasis in males (85.9%) was aligned with previous reports [39]. This suggests a consistent pattern of gender distribution of schistosomiasis cases in Saudi Arabia. Such discrepancy could most likely be the result of the difference in contact probability of individuals with the infective stage (cercaria). Indeed, during their agricultural activities, men are in close contact with fresh water and thereby more exposed to the transcutaneous transmission of the parasite.

Despite the fact that school-aged children are more vulnerable to infection, our results revealed that younger individuals (15–39 years old) are the most prevalent group (75.5%) with schistosomiasis while almost 15.4% of cases were in the 5–14 year age group. These findings are consistent with a previous study conducted by Mohammad (2014), who reported that the 15–44 year age group had the highest infection rate [39]. Such a finding is not surprising if we consider that schistosomiasis in KSA is due to post-transmission (as most of the immigrants came as workers within the age of 20–40 years old) rather than due to being infected in the country. 

Finally, we acknowledge that relaying solely on microscopic examination of stool/urine samples for the diagnosis of schistosomiasis has a significant limitation. Although this classic diagnostic method has historically been used to detect *Schistosoma* eggs, it is limited by its insufficient sensitivity. However, the reported findings remain of great importance to draw the attention of health authorities and policy makers on the urgent need for integrated control strategies to curtail this health issue.

## 5. Conclusions

Despite the marked decline of schistosomiasis in KSA, it is still reported in both clinical forms (intestinal and urinary schistosomiasis). However, while schistosomiasis is almost absent in the northern and central regions, schistosomiasis is more frequent in the western and south-western regions of the country. Males, expatriates and younger individuals are the most at-risk groups. *Schistosoma* cases in KSA are more likely a post-transmission infection due to imported infection by immigrant movements from endemic countries. Therefore, effective surveillance to screen immigrants, visitors and returning international travelers from endemic areas for *Schistosoma* is crucial to monitor this dangerous disease. Collectively, these findings draw attention to the critical role of international movements and demographic factors in sustaining schistosomiasis in non-endemic regions such as KSA, highlighting the need for comprehensive, cross-disciplinary interventions based on the One Health paradigm to address the region’s persistent schistosomiasis challenge. Such an approach has to address the complex interaction between human, animal and environment. Interventions should include (i) a monitoring program for the detection of the parasite in human and animal hosts, (ii) a management strategy of the freshwater resources to control snails, (iii) a program for community large-scale treatment and education to raise awareness and enhance capacities for human health and (iv) a plan to develop the capacity of health professional, veterinarians, and environmental scientists to work on comprehensive control strategies. Overall, our study contributes to the comprehensive understanding of schistosomiasis in KSA, providing a foundation for evidence-based policies and interventions.

## Figures and Tables

**Table 1:** Annual distribution of schistosomiasis in KSA (2014–2020).

Year	Total Population	Number of Examined	Positive n (%)	Negative n (%)	Incidence/100,000 Population	*p* Value	OR(95%CI)
2014	28,309,273	787,699	117 (0.0148)	787,582 (98.6)	0.413	<0.001	1.41 (0.98–2.03) ^ns^
2015	29,816,382	781,366	159 (0.0203)	781,207 (97.97)	0.533		1.93 (1.36–2.74) ***
2016	30,954,198	726,216	119 (0.0164)	726,097 (98.36)	0.384		1.56 (1.08–2.23) **
2017	30,977,355	593,011	103 (0.0174)	592,908 (98.26)	0.332		1.65 (1.14–2.38) **
2018	30,196,281	587,608	96 (0.0163)	587,512 (98.37)	0.318		1.55 (1.07–2.25) *
2019	30,063,799	525,301	47 (0.0089)	525,254 (99.11)	0.156		0.85 (0.56–1.29) ^ns^
2020	31,552,510	370,280	39 (0.0105)	370,241 (98.95)	0.123		Reference
Total	-	4,371,481	680 (0.0155)	4,370,801 (98.45)	2.155		-

* *p* value < 0.05; ** *p* value < 0.01; *** *p* value < 0.001; and ^ns^ not significant.

**Table 2:** Regional distribution of schistosomiasis in KSA (2014–2020).

Regions	Population	Number of Examined	Positive Cases	Cumulative Incidence per 100,000	OR(95%CI)
Mecca	9,261,257	1,026,438	420	4.54	5.57 (2.49–12.47) ***
Medinah	2,291,092	682,426	51	2.22	1.02 (0.44–5.36) ^ns^
Aseer	2,354,320	278,936	47	1.59	2.29 (0.98–1.93) *
Jazan	1,670,569	554,056	70	4.19	1.72 (0.75–3.96) ^ns^
Al-Bahah	506,866	454,227	83	16.4	2.49 (1.09–5.69) *
Eastern region	3,485,383	81,673	6	0.17	0.09 (0.02–0.39) ***
Najran	621,040	419,683	3	0.49	Reference
Total	26,550,777	4,371,481	680	2.56	-

* *p* value < 0.05; *** *p* value < 0.001; and ^ns^ not significant.

**Table 3:** Type of Schistosomiasis among different regions in KSA (2014–2020).

Regions	Type of Schistosomiasis		Total n (%, Out of All Positive Cases)	*p* Value
	Urinary n (%)	Intestinal n (%)		
Mecca	22 (3.2)	398 (58.5)	420 (61.7)	<0.001
Medinah	11 (1.6)	40 (5.9)	51 (7.5)	
Aseer	27 (4.0)	20 (3.0)	47 (7.0)	
Jazan	69 (10.1)	1 (0.15)	70 (10.3)	
Najran	2 (0.29)	1 (0.15)	3 (0.4)	
Al-Bahah	3 (0.44)	80 (11.8)	83 (12.2)	
Eastern region	5 (0.74)	1 (0.15)	6 (0.9)	
Total	139 (20.4)	541 (79.6)	680 (100)	

**Table 4:** Gender distribution of people with Schistosomiasis in the different regions of KSA (2014–2020).

Regions	Gender		Total n (%, Out of All Positive Cases)	*p* Value
	Male n (%)	Female n (%)		
Mecca	371 (54.5)	49 (7.2)	420 (61.7)	<0.001
Medinah	44 (6.5)	7 (1.0)	51 (7.5)	
Aseer	42 (6.2)	5 (0.7)	47 (7.0)	
Jazan	52 (7.6)	18 (2.6)	70 (10.3)	
Najran	3 (0.44)	0	3 (0.4)	
Al-Bahah	68 (10)	15 (2.2)	83 (12.2)	
Eastern region	4 (0.59)	2 (0.3)	6 (0.9)	
Total	584 (85.9)	96 (14.1)	680 (100)	

**Table 5:** Distribution of schistosomiasis according to nationality among KSA regions (2014–2020).

Regions	Nationality		Total n (%, Out of All Positive Cases)	*p* Value
	Saudi n (%)	Expatriates n (%)		
Mecca	87 (12.7)	333 (49)	420 (61.7)	<0.001
Medinah	0	51 (7.5)	51 (7.5)	
Aseer	16 (2.3)	31 (4.6)	47 (7.0)	
Jazan	15 (2.2)	55 (8.1)	70 (10.3)	
Najran	0	3 (0.44)	3 (0.4)	
Al-Bahah	78 (11.5)	5 (0.7)	83 (12.2)	
Eastern region	4 (0.6)	2 (0.3)	6 (0.9)	
Total	200 (29.4)	480 (70.6)	680 (100)	

**Table 6:** Distribution of schistosomiasis among different age groups in KSA regions (2014–2020).

Regions	Age Groups				Total n (%, Out of All Positive Cases)	*p* Value
	<5 Years n (%)	5–14 Years n (%)	15–39 Years n (%)	>40 Years n (%)		
Mecca	0	44 (6.5)	335 (49.2)	41 (0.6)	420 (61.7)	<0.001
Medinah	0	0	44 (6.6)	7 (1.0)	51 (7.5)	
Aseer	1 (0.15)	19 (2.8)	26 (3.8)	1 (0.15)	47 (7.0)	
Jazan	0	14 (2.1)	53 (7.8)	3 (0.44)	70 (10.3)	
Najran	0	0	3 (0.4)	0	3 (0.4)	
Al-Bahah	0	28 (4.1)	46 (6.8)	9 (1.3)	83 (12.2)	
Eastern region	0	0	6 (0.9)	0	6 (0.9)	
Total	1 (0.1)	105 (15.4)	513 (75.5)	61 (9.0)	680 (100)	

## Data Availability

The data was were extracted from the General Authority for Statistics (GASTAT).
